# The Relationship between Objectively Measured and Self-Reported Sedentary Behaviours and Social Connectedness among Adolescents

**DOI:** 10.3390/ijerph16020277

**Published:** 2019-01-18

**Authors:** Lauren Arundell, Jo Salmon, Jenny Veitch, Anna Timperio

**Affiliations:** Institute for Physical Activity and Nutrition (IPAN), School of Exercise and Nutrition Sciences, Deakin University, Geelong 3220, Australia; jo.salmon@deakin.edu.au (J.S.); jenny.veitch@deakin.edu.au (J.V.); anna.timperio@deakin.edu.au (A.T.)

**Keywords:** adolescents, youth, social connectedness, sitting, sedentary

## Abstract

Adolescents spend significant amounts of time engaged in various types of sedentary behaviour (SB). This study examined associations between adolescents’ objectively measured sedentary time, sitting time, specific self-reported SBs and social connectedness. Adolescents (*n* = 429, 15.5 years, 41% male) completed an online survey reporting time in seven SBs (TV/videos/DVDs, computer/video games, internet, homework, reading, car and bus travel; examined individually and summed for screen time and total SB), and social connectedness using the eight-item Social Connectedness Scale. A subsample (*n* = 353) also wore an ActiGraph GT3X+ (model GT3X+, Pensacola, FL, USA) accelerometer to measure sedentary time (<100 cpm) and *n* = 237 wore an *activ*PAL (PAL Technologies Ltd., Glasgow, Scotland) inclinometer to measure sitting time. Multiple linear mixed models determined associations between each SB variable and social connectedness, adjusting for confounders. Adolescents spent on average 7.8 h/day in self-reported total SB, 4.4 h/day in screen time, 9.1 h/day in ActiGraph-measured sedentary time, and 9.5 h/day in *activ*PAL-measured sitting time. After adjusting for age, sex and area level socioeconomic status, total SB (−0.24, 95%CI: −0.37, −0.11), screen time (−0.23, 95%CI: −0.41, −0.05) and two individual SBs (computer/video games (−1.07, 95%CI: −1.53, −0.60), homework (−0.61, 95%CI: −1.04, −0.18) were negatively associated with social connectedness. There were no associations with the objective measures. The relationships may be bi-directional; therefore, future research should involve longitudinal designs and explore other potential contributing factors.

## 1. Introduction

Sedentary behaviours, defined as sitting or lying behaviours that require minimal energy expenditure (<1.5 METs) [1], make up the majority of adolescents’ waking hours, particularly during school- (e.g., sitting during class time) and leisure-time (e.g., TV viewing, digital tablet use, internet and social media use) [2,3]. Australian and international recommendations state that children and adolescents (5–18 year olds) should limit their recreational screen-based sedentary behaviour to less than two hours per day and reduce and interrupt prolonged periods of sitting as often as possible [4,5]. However, compliance with screen time recommendations is poor among Australian children (36% of 5–11 year olds) and even lower among adolescents (21% of 12–17 year olds) [6].

Sedentary behaviours are associated with negative health outcomes among children and youth [7]. However, this evidence is mixed depending on whether studies have explored health associations with specific sedentary behaviours (particularly screen time) or with total objectively-assessed sedentary or sitting time [8]. A recent systematic review found children and youth (5–17 years) with higher screen-time showed more hyperactivity/inattention problems, lower psychological well-being and perceived quality of life, and greater internalizing of problems [9]. The authors did, however, call for additional research using objective measures of sedentary behaviour to substantiate these findings. An earlier review which focused on TV viewing also found that children and youth (5–17 years) who watched less TV were more emotionally stable and outgoing [7]. In addition to the “traditional” technologies (i.e., TV viewing), the prevalence of “new” technologies [10] (i.e., digital tablets, smart phones, game consoles, readily available internet access) have changed the way in which adolescents communicate and connect with others [11] For example, in 2014–2015, 99% of Australian adolescents, aged 15–17 years accessed the internet at home during a typical week and 91% did so for social networking [12]. In comparison, ten years prior to this, only 56% of Australian households had internet access [13]. Furthermore, the proportion of adolescents using the computer, internet and game consoles increased substantially between 2009–2012 [12]. Sedentary behaviours and the transformation in the availability of technology that promotes sitting may also influence how connected adolescents feel socially. For example, the increase in smartphone use among adolescents has led to an increase in online communication with peers and online communities [14], however it is also associated with a decrease in participation in real life social interactions [14] which may impact social connectedness.

Social connectedness has been defined as an “enduring interpersonal closeness with the social world” [15] (page 316). A person’s social connectedness develops in childhood (e.g., parent and child connections) and evolves throughout adolescence and into adulthood. People who do not feel socially connected often find interacting with others difficult, which may result in a solitary life [16]. Adolescents aged 13–14 years who do not feel connected to others are less likely to complete school, are more likely to experience anxiety/depressive factors [17,18] and engage in risk taking behaviours in later adolescence (i.e., drinking, smoking and drug use) [17]. Longitudinal data from over 1000 participants in the Dunedin Multidisciplinary Health and Development Study (DMHDS) showed that social connectedness during late adolescence (15–18 years) is a stronger predictor of wellbeing in adulthood (32 years) than academic achievement [19].

As adolescence is a period of rapid emotional and social change, coupled with elevated sedentary behaviour [6], it is important to understand the relationships between sedentary behaviour and wellbeing outcomes such social connectedness that could have detrimental effects on wellbeing in later years [20]. Establishing associations between participation in specific sedentary behaviours, some of which may be more socially engaging than others (e.g., traveling by bus with peers vs travelling in a car with one parent vs completing homework on their own) and social connectedness may also inform the development of interventions. The aim of this study was to examine and explore the relationships between objectively-assessed total sedentary and sitting time, self-reported specific and summed sedentary behaviours, and social connectedness among adolescents.

## 2. Materials and Methods

### 2.1. Sample

This cross-sectional sample is drawn from the baseline data of the NEArbY study, collected in 2014–2015, which has been described elsewhere [21]. Briefly, Secondary schools (*n* = 137) selected from four strata within Level 1 Statistical Areas of Melbourne (based on objectively-assessed walkability [22] and area-level income [23]: high income/high walkability; high income/low walkability; low income/high walkability; and low income/low walkability) were invited. A total of 18 schools participated in the NEArbY study (response rate 14%). Students in classes selected by each school were invited to attend a recruitment presentation at the school and recruitment packs were distributed to interested students. Completion of an online survey and wearing an ActiGraph accelerometer (model GT3X+, Pensacola, FL, USA) were core components of the study; participants could also opt to wear an *activ*PAL inclinometer (PAL Technologies Ltd., Glasgow, Scotland). Overall, 1454 recruitment packs were delivered within the 18 schools. Repeat visits to some schools occurred and the number of recruitment packs delivered during one of these visits was not recorded. The final number of recruitment packs delivered is therefore unable to be reported.

### 2.2. Ethics

Ethical approval was received from the Deakin University Human Ethics Advisory Group (HEAG-H 152_2013), the Department of Education and Training (2013_002182) and the Catholic Education Office (Project ID #1950). The School Principal provided written consent for the students at their school to be invited to participate. Written parental consent and student assent was required.

### 2.3. Measures

#### 2.3.1. Objectively Measured Sedentary and Sitting Time

Sedentary time was assessed using ActiGraph Accelerometers (model GT3X+, Pensacola, FL, USA). Participants were fitted with the ActiGraph during school time by trained research staff. Participants were asked to wear the device on the right hip on an elastic belt for eight days during waking hours and remove it for all water-based activities. ActiGraph have been shown to have acceptable validity for determining sedentary time among youth [24]. Data were downloaded in 15s epochs using Actilife Lifestyle Monitoring System, Version 5.1.

Sitting time was concurrently assessed using the *activ*PAL^TM^ inclinometer (PAL Technologies Ltd., Glasgow, Scotland). The inclinometer function classifies an activity into periods of sitting/lying, standing and stepping [25] and has acceptable validity in adolescents [26]. Participants were fitted with the *activ*PAL^TM^ during school time by trained researchers. Participants were asked to wear the *activ*PAL^TM^ during waking hours for eight days on an elastic garter positioned at the mid-anterior aspect of the right thigh and removed during water-based activities.

#### 2.3.2. Subjectively Measured Sedentary Behaviours

To assess time spent in specific sedentary behaviours, adolescents completed an online survey (hosted by Qualtrics) during class time under the guidance of trained researchers. Adolescents were asked to indicate how much time during a typical weekday and weekend day they spent in seven sedentary behaviours outside school hours: Watching TV, videos or DVDs; playing sedentary computer or video games (e.g., Nintendo or Xbox); using the internet, emailing or other electronic media for leisure (e.g., Facebook, Instagram); doing homework (including reading, writing or using the computer); reading a book or magazine not for school (including comic books); riding in a car; and riding in a bus. The response options (and coding in parentheses) were: None (0); 15 min/day (0.25); 30 min/day (0.5); 1 h/day (1); 2 h/day (2); 3 h/day (3); or 4+ h/day (4). Data relating to concurrent participation was not collected. Overall sedentary behaviour time was calculated by summing time spent in each of the seven behaviours. Screen time was calculated by summing time spent watching TV, videos or DVDs, playing sedentary computer or video games and using the internet. The responses for a weekday were multiplied by five, the responses for a weekend day were multiplied by 2, and these were then summed [27] and divided by 7 to compute the average hours/day in each sedentary behaviour. The survey items were adapted from previously published survey items [28,29] and have shown acceptable reliability (intraclass correlation coefficient 0.77) [29] and validity amongst youth compared to heart rate [28] and accelerometery [28,29].

#### 2.3.3. Social Connectedness

The online survey also included the Social Connectedness Scale, an eight item measure with a previously established internal reliability of 0.91 [16]. Using a four-point Likert scale, adolescents were asked to report their agreement (strongly disagree to strongly agree) with the following statements: *I feel disconnected to the world around me; even around people I know, I don’t feel that I really belong; I feel so distant from people; I have no sense of togetherness with my peers; I don’t feel related to anyone; I catch myself losing all sense of connectedness with society; even among my friends, there is no sense of brother/sisterhoods; and I don’t feel that I participate with anyone or any group*. All responses were reverse coded and summed to create an overall social connectedness score (range 8–32), where a higher score reflects a higher level of social connectedness. The scale had acceptable internal consistency in the current dataset (Cronbach’s alpha = 0.94).

#### 2.3.4. Socio-Demographics

Adolescents’ age and sex were collected from the adolescent survey. Area-level socio-economic status (SES) was obtained by matching participant residential postcodes with data from the 2011 Australian Census of Population and Housing: Socio-Economic Indexes for Areas (SEIFA) [30]. The IRSAD (Index of Relative Socio-economic Advantage and Disadvantage) was used as it provides information about the people and households within an area and includes measures of both advantage and disadvantage.

### 2.4. Data Management and Analysis

The raw ActiGraph and *activ*PAL data were analysed using specifically developed excel macros and Stata code (Stata 12). Non-wear time was considered 60 consecutive minutes of zero counts for the ActiGraph [31]. The same criteria was applied to the *activ*PAL using data from vertical (accelerometer) axis of that device. Participants were required to have >8 h of valid data on at least 3 weekdays and >7 h of valid data on at least one weekend day to be included in analyses [32]. Sedentary time was defined as <100 counts.min^−1^ (<25 counts per 15 s epoch) which is the cut-off point that most closely represents sitting time among children [33]. To align with the objective measures, average daily duration of each self-reported sedentary behaviour and the summed variables was computed. All outcomes were tested for normality. Weekly time watching TV, computer/video games, reading and riding in a car and bus were skewed and transformed (square root). Differences in sex (via chi-square tests), age, social connectedness and SES (via *t*-tests) between adolescents with and without ActiGraph data or *activ*PAL data were assessed. Given the opt-in nature of the measures, differences between ActiGraph measured sedentary time between adolescents with and without *activ*PAL-measured sitting data, and differences in *activ*PAL measured sitting time between participants with and without ActiGraph-measured sedentary time were examined via *t*-tests. Differences in self-reported sedentary time between those with and without valid ActiGraph and *activ*PAL data were also examined (via *t*-test, exact *p*-values reported).

Separate mixed models were used to analyse associations between each independent variable (sedentary time, sitting time and each specific sedentary behaviour) and social connectedness. The first model assessed the unadjusted relationship which for the objective measures, included adjusting for wear time. The second model included age, sex, SES and monitor specific wear time as potential confounders. It is important to determine associations between sedentary time, sitting time and social connectedness independent of moderate- to vigorous-intensity physical activity (MVPA). Therefore, ActiGraph measured sedentary time analysis were also adjusted for MVPA (defined as ≥4 METS calculated using age-specific cut-points [34]); and *activ*PAL measured sitting analysis were adjusted for stepping time, to determine the relationship between these outcomes and social connectedness, independent of physical activity [35]. School was treated as a random effect in all analysis and significance was set at *p* < 0.05. To determine local effect size, Cohens *f*^2^ were calculated on raw data, and a value of *f*^2^ ≥ 0.02, *f*^2^ ≥ 0.15, and *f*^2^ ≥ 0.35 represented a small, medium, and large effect sizes, respectively [36].

## 3. Results

Informed consent was received for 528 participants to provide survey and accelerometer data and 387 provided consent to wear an *activ*PAL. In total, 445 participants completed the survey and 429 provided data for all variables required for the current analysis (81% of consenting participants). Of these, 353 had valid ActiGraph data (66.8% of consenting participants), 237 had valid *activ*PAL data (61% of consenting participants) and 222 had both valid ActiGraph and *activ*PAL data. Participants were on average 15 years of age, 41% were male and their social connectedness score was 28 (range 8–32) (Table 1). Participants with valid ActiGraph data were slightly younger than those without valid ActiGraph data (15.3 ± 1.57 years vs 15.9 ± 1.56 years, *p* < 0.05) and had higher SES (1007.6 ± 4.5 IRSAD score vs 970.5 ± 11.5 IRSAD score, *p* < 0.05). There were no differences according to sex or social connectedness. There were no significant differences in age, sex, social connectedness or SES between participants with and without valid *activ*PAL data. There were no significant differences in ActiGraph-measured sedentary time between participants who did (*n* = 222) or did not (*n* = 131) also provide *activ*PAL-measured sitting data, nor were there differences in *activ*PAL measured sitting time between participants who did (*n* = 222) or did not (*n* = 15) also provide ActiGraph measured sedentary time. There were no significant differences in self-reported sedentary time between those with and without valid ActiGraph and *activ*PAL data. Therefore, to maximise the sample size, all participants with available valid data from the different devices were included in the analysis.

The average daily duration of each sedentary behaviour and total sedentary and sitting time is shown in Table 2. On average, adolescents spent more than 9 h in ActiGraph-measured sedentary time and *activ*PAL-measured sitting and reported almost 8 h of self-reported sedentary behaviours per day. Adolescents reported spending the most time engaged in internet use, followed by TV viewing, homework, computer/video games, travelling in a car, reading and travelling in a bus.

Table 3 shows the unadjusted and adjusted associations between sedentary time, sitting time and self-reported sedentary behaviours and social connectedness and effect sizes. In the fully adjusted model, self-reported total sedentary time, screen time, and time spent on computer/video games and homework were associated with less social connectedness. For example, for each additional hour per day spent playing computer/video games, there was a corresponding 1.07 unit reduction in social connectedness.

## 4. Discussion

This study found inverse associations between self-reported out-of-school sedentary time, screen time and two specific self-reported sedentary behaviours and social connectedness. No significant associations were found with the objective measures of daily sitting or sedentary time, which suggest that it is the specific behaviours that adolescents are engaging in that may be important for their levels of social connectedness. Findings provide additional evidence that supports the need for interventions to reduce adolescents’ participation in sedentary behaviours, particularly screen time and computer/video games. Reductions in these sedentary behaviours may subsequently reduce their risk of many other psycho-social outcomes associated with these behaviours such as poor psychological wellbeing [9] and behavioural conduct [37] as well as the negative health outcomes associated with low social connectedness [17,18].

Of the individual sedentary behaviours assessed, computer/video game use showed the strongest negative association with social connectedness, despite adolescents spending more time watching TV, using the internet and completing homework. Although co-participation data were not collected in the current study, the association may be because computer/video gaming is likely to be performed on one’s own, and if interactions with others do occur, they are often done via online multiplayer functions which may not have the same social benefits as playing computer/video games together in person. Future research should tease out the distinction between playing such games alone, in the personal company of others, or alone but on online platforms that allow verbal communication between players/friends, since these different modes of play may lead to different outcomes. Elevated video game use during adolescence has also been shown to be associated with other indicators of poor mental health such as unfavourable behavioural conduct, anti-social behaviour [33] and depressive symptoms [38]. However, opportunities to engage in computer/video games are increasing with over half of 12–17 year olds in Australia having a computer in their bedroom and 29% having a video game console in 2011–2012 [6], and this is likely to have increased since then. In addition, computer and video game play are no longer restricted to those with a console as they can be played on smart phones and digital tablets, which are owned by 89% of Australian adolescents [39]. Given the increasing use of smartphones and digital technology, future research should explore relationships between smartphone and digital tablet use and social connectedness.

The negative relationship between homework and social connectedness may be attributed to the increasing amount of homework that adolescents are required to complete as they move to more senior year levels in school. As students near the end of their schooling, they are expected to perform at least 1–3 h of homework per night and up to 6 h over the weekend [40]. Homework is also typically completed in isolation and may displace opportunities for adolescents to participate in activities where they engage with and feel connected to others. As homework is an important requirement associated with academic outcomes [33], it may not be possible to reduce time spent doing homework but interventions may consider replacing individual homework tasks with group or team orientated tasks. It also highlights the need to ensure social opportunities occur during leisure time.

Interestingly, no association was found between TV viewing time and social connectedness, despite previous studies showing TV viewing to be associated with unfavourable behavioural conduct and anti-social behaviour [7,37]. This may be due to a number of reasons; firstly, adolescents may be engaging in screen-stacking, where a person uses multiple screens at once which predominantly occurs while watching TV [41], and therefore their main attention is elsewhere. This was not captured in the current study but may be an important consideration for future survey development. Alternatively, participants may have been watching TV with others or they may have been interrupting their viewing with social interaction (e.g., talking to family or friends).

The relationships between individual sedentary behaviours and social connectedness do not appear to be dependent on the time spent in these behaviours, as adolescents in the current study spent almost twice as long watching TV and using the internet as they did playing computer/video games. However, there may be additional factors not measured in this study that contribute to social connectedness, such as co-participation in these behaviours with others (in person, remotely or on-line), the size and quality of their friendship networks, the content of the TV shows watched and computer/video games played, time spent in the neighbourhood or outdoors, and the extent of engagement in social activities outside of school (e.g., sport, scouts, informal catch-ups with friends).

The lack of associations between objectively-assessed sitting and sedentary time with social connectedness may be attributed to the nature of these measures. *Activ*PALs and ActiGraphs measure time spent sitting and sedentary, respectively, and are therefore unable to differentiate between time spent in social (e.g., sitting and talking) and non-social (reading) sitting/sedentary behaviours, which are more readily captured via surveys. These inconsistencies concur with the findings from a systematic review by Cliff and colleagues who found mixed evidence of associations between sedentary behaviours, sedentary time and health outcomes among youth [8]. However, the self-report items used to assess individual sedentary behaviours in the current study also did not capture the social context of the activity. There is a need for both objective and subjective measures to capture sedentary behaviour engagement as well as the social context in which they are performed.

In acknowledging the findings of the current study, it is important to consider that the relationship between sedentary behaviours and social connectedness may be bi-directional. Adolescents who engage in high levels of sedentary behaviour may feel less connected to the outside world and be more likely to have poorer social connectedness and engage in behaviours that can be performed alone. Conversely, adolescents who feel less connected may fill their time with particular sedentary behaviours. Future longitudinal research is required to identify the strength and direction of this relationship. Further, the small effect sizes are indicative of this exploratory investigation and the need for replication to extend the findings.

A major strength of this study was the use of both objective and self-report measures of sedentary behaviour. This enabled associations with an overall measure of sedentary and sitting time, as well as time spent in individual sedentary behaviours, to be assessed. However, it is possible that some of the sedentary behaviours that were reported were performed simultaneously, which may have resulted in over-estimations of total sedentary behaviour and screen time. The selected self-report measures also did not distinguish whether the behaviour was performed while sitting or with others, and the list was limited to just seven sedentary behaviours outside school hours and therefore may have missed other sedentary behaviours that contribute to social connectedness. Further, although the greatest differences between adolescents’ levels of social interaction are likely to occur during their discretionary time, adolescents may also experience varying levels of social interaction during school hours. The current measures did not distinguish sedentary behaviour that did or didn’t involve social interaction nor differentiate sedentary behaviours and social connectedness that occurred during, or out of school hours. It is important that future studies aiming to further explore the relationships between sedentary behaviours and social connectedness, update their self-reported behaviour measures to include options in line with advancing technologies, include more contextualised measures of sedentary behaviour, consider multi-tasking factors, sedentary behaviours and social interactions within the school day, which may moderate the relationship between sedentary behaviour and social connectedness. Although the social connectedness scale is reliable, future studies may consider an objective and real-time measure of social interaction such as ecological momentary assessment (EMA) which may also provide valuable contextual information [42].

## 5. Conclusions

This study found that overall self-reported sedentary behaviour outside school hours and screen time and time spent in computer/video game use and homework specifically was associated with lower levels of social connectedness. Given the risks associated with both high levels of sedentary behaviour and low levels of social connectedness, intervention strategies that encourage adolescents to sit less and engage in more social opportunities during leisure time are required. However, future research using a wide variety of emerging sedentary behaviour options is needed, as is longitudinal research to identify temporal relationships between these variables.

## Figures and Tables

**Table 1:** Participant demographics (*n* = 429).

Characteristic	Mean (±SD)/%
Age, mean years (±SD)	15.5 (±1.6)
Sex: % boys	41%
IRSAD ^α^, mean (±SD; range 488–1148)	1002.14 (±89.95)
Social connectedness scale, mean value (±SD; range 8–32) ^b^	27.87 (±4.84)

^α^ Index of Relative Socio-economic Advantage and Disadvantage; ^b^ higher score denotes higher level of social connectedness.

**Table 2:** Mean (±SD) duration (hours/day) of objectively measured daily sedentary, daily sitting time and self-reported sedentary behaviours outside school hours.

Behaviour	*N*	Mean Duration Hours/Day (±SD)
**Objectively-measured**		
Sedentary time^a^	353	9.12 (±1.52)
ActiGraph accelerometer wear time ^a^	353	13.28 (±1.47)
Sitting time ^b^	237	9.53 (±1.57)
***activ***PAL inclinometer wear time ^b^	237	13.78 (±1.43)
**Self-reported**		
Total sedentary behaviour	429	7.75 (±3.54)
Screen-time	429	4.43 (±2.58)
Internet	429	2.07 (±1.33)
TV	429	1.54 (±1.15)
Homework	429	1.52 (±1.09)
Computer/video games	429	0.81 (±1.13)
Car	429	0.76 (±0.74)
Reading	429	0.69 (±0.96)
Bus	429	0.35 (±0.60)

^a^ ActiGraph accelerometer derived sedentary time; ^b^ *activ*PAL incolinometer derived sitting time.

**Table 3:** Associations between objectively-measured daily sedentary and sitting time, self-reported sedentary behaviours outside school hours (hours/day) and social connectedness.

Independent Variables	Unadjusted			Adjusted ^¥^			
	COEFF.	95% CI	*p* Value	COEFF.	95% CI	*p* Value	Effect Size (Cohens *f*^2^)
**Objectively-measured behaviours (hours/day)**							
Sedentary time	−0.24 ^a^	−0.77, 0.29	0.377	0.05 ^a^	−0.59, 0.70	0.859	0.04
Sitting time	−0.39 ^b^	−0.85, 0.61	0.090	−0.25 ^b^	−0.84, 0.35	0.418	<0.01
**Self−reported behaviours (hours/day)**							
Total sedentary behaviour	−0.26	−0.38, −0.13	**<0.001**	−0.24	−0.37, −0.11	**<0.001**	<0.01
Screen time	−0.20	−0.37, −0.02	**0.032**	−0.23	−0.41, −0.05	**0.013**	0.02
Internet	−0.39	−0.74, −0.5	**0.026**	−0.30	−0.65, 0.5	0.096	0.01
TV	−0.05	−0.44, 0.35	0.940	0.02	−0.41, 0.37	0.906	0.01
Homework	−0.65	−1.07, −0.23	**0.002**	−0.61	−1.04, −0.18	**0.005**	0.01
Computer/video games	−0.63	−1.03, −0.22	**0.003**	−1.07	−1.53, −0.60	**<0.001**	0.04
Car	−0.26	−0.88, 0.34	0.403	−0.20	−0.82, 0.41	0.516	<0.01
Reading	−0.51	−0.99, 0.04	**0.034**	−0.41	−0.88, 0.07	0.095	<0.01
Bus	−0.39	−1.14, 0.37	0.318	−0.17	−0.93, 0.59	0.665	0.01

Results of mixed models; all models adjusted for clustering by school; ^a^ Model adjusted for ActiGraph wear time and MVPA; ^b^ Model adjusted for *activ*PAL wear time and stepping; ^¥^ adjusted for age, sex and SES. Significance is noted by bold text.
